# Ethnicity, deprivation, and the use of patient portals in England’s general practices 2018-2020

**DOI:** 10.1093/eurpub/ckac129.302

**Published:** 2022-10-25

**Authors:** A Alturkistani, G Greenfield, T Beaney, J Norton, CE Costelloe

**Affiliations:** 1 Department of Primary Care and Public Health, Imperial College, London, UK; 2 National Institute for Health Research, Applied Research Collaboration Northwest London, London, UK; 3 Division of Clinical Studies, Institute of Cancer Research, London, UK

## Abstract

**Background:**

Patient portals are made available and widely promoted in healthcare systems in the USA and Europe. These technologies can help patients access healthcare, receive timely treatment, and manage their health through services such as appointment booking and repeat prescription ordering. However, it is not clear if all patients who need the services are using them. This study explored patient portal use (online appointment booking and repeat prescription ordering features) and patient characteristics among NHS England GP practice patients.

**Methods:**

The study used cross-sectional participant-level data from the GP Patient Survey (GPPS) of 2018, 2019, and 2020. Performing multilevel regression analysis, we explored the association between patient portal feature use and ethnicity and deprivation and controlled for eight other patient characteristics and one GP practice level characteristic, and modelled GP practice as a random effect in the model.

**Results:**

In the fully adjusted model controlled for all patient characteristics and GP characteristics, participants of the Black and Other ethnic groups were less likely to have used online appointment booking (OR: 0.84, 95% CI:0.81, 0.86, and OR: 0.96, 95% CI: 0.92, 0.99, respectively) and online repeat prescription ordering (OR: 0.76, 95% CI: 0.74-0.78 and OR: 0.78, 95% CI: 0.75-0.81, respectively) compared to the White ethnic group. Association with patient portal use increased proportionally with reduced deprivation ranking.

**Conclusions:**

In NHS England GP practices, certain ethnic minority groups and high deprivation ranking is associated with a reduced likelihood of using patient portals. If patient portals are the only route to access services, it is likely to lead to inequalities in use by some patient groups introducing unfair access to the services. Patients could continue to be provided with alternatives to patient portals to prevent potential inequities in access to services.

**Key messages:**

• Patient portals are widely used in the healthcare system and can benefit all patients given that disparities are prevented by understanding patient groups who cannot access portals.

• Understanding patient groups less likely to use patient portals could help adapt healthcare system services and meet the needs of all patient groups.

